# *daf-2* modulates regeneration of mechanosensory neurons II

**DOI:** 10.17912/W2SM1T

**Published:** 2017-12-01

**Authors:** Zehra C. Abay, Michelle Yu-Ying Wong, Brent Neumann

**Affiliations:** 1 Neuroscience Program, Monash Biomedicine Discovery Institute and Department of Anatomy and Developmental Biology, Monash University, Melbourne VIC 3800, Australia

## Description

**​**The *daf-2* gene encodes an insulin-like growth factor/IGF-1 receptor that regulates *C. elegans* embryonic and larval development. It has previously been shown that DAF-2 inhibits neurite regeneration of the GABAergic motor neurons and PVD sensory neurons in an age-dependent fashion (Bryne et al., 2014; Kravtsov et al., 2017). Following injury, the posterior lateral microtubule (PLM) neurons are capable of regenerating through axonal fusion, a highly efficient regrowth mechanism in which separated fragments fuse back together (Ghosh-Roy et al., 2010; Neumann et al., 2011; Neumann et al. 2015; Abay et al., 2017). We previously established that a critical event for axonal fusion to occur is the exposure of injury-induced phosphatidylserine (PS) ‘save-me’ signals (Neumann et al., 2015). The level of PS exposed increases with advancing age (Abay et al., 2017). To determine if *daf-2* is involved in this age-dependent modulation of PS exposure, we visualised and quantified the level of PS exposed after PLM axotomy using a secreted, tagged version of Annexin V (Neumann et al., 2011; Mapes et al., 2012; Neumann et al. 2015). Mutation of *daf-2* had no effect on PS exposure 1 h post-axotomy, with no significant differences observed on either the distal or proximal axon segments (Table 1).​

**Table 1.** Quantification of the relative level of PS exposed 1 h post-axotomy.

**Table:** 

Genotype	PS exposed on distal axon (relative to pre-axotomy)	n	PS exposed on proximal axon (relative to pre-axotomy)	n
wild-type	1.53 ± 0.105	28	1.44 ± 0.0855	28
*daf-2(e1370)*	1.51 ± 0.167	26	1.57 ± 0.166	26

## Reagents

One-day-old adult hermaphrodites were used for all experiments, and were grown under standard conditions at 20°C. The BXN301 [*daf-2(e1370); smIs95(Phsp-16.2::sAnxV::mRFP); zdIs5(Pmec-4::GFP)*] strain was used along with the CU4204 [*smIs95(Phsp-16.2::sAnxV::mRFP); zdIs5(Pmec-4::GFP)*] control strain. The *daf-2(e1370)* allele has been considered temperature sensitive for the dauer phenotype, but not for the long-lived phenotype. At 20°C, *daf-2(e1370)* animals display a greater than 2-fold increase in lifespan compared to the wild-type (Kenyon et al., 1993). Laser axotomy, microscopy and quantification of data was performed as previously described (Abay et al 2017).
